# Chaos and dynamical complexity in the quantum to classical transition

**DOI:** 10.1038/s41598-018-20507-w

**Published:** 2018-02-01

**Authors:** Bibek Pokharel, Moses Z. R. Misplon, Walter Lynn, Peter Duggins, Kevin Hallman, Dustin Anderson, Arie Kapulkin, Arjendu K. Pattanayak

**Affiliations:** 1 Department of Physics and Astronomy, Carleton College, Northfield, Minnesota 55057 USA; 2 128 Rockwood Cr, Thornhill, Ont L4J 7W1 Canada

## Abstract

We study the largest Lyapunov exponents *λ* and dynamical complexity for an open quantum driven double-well oscillator, mapping its dependence on coupling to the environment Γ as well as effective Planck’s constant *β*^2^. We show that in general *λ* increases with effective Hilbert space size (as *β* decreases, or the system becomes larger and closer to the classical limit). However, if the classical limit is regular, there is always a quantum system with *λ* greater than the classical *λ*, with several examples where the quantum system is chaotic even though the classical system is regular. While the quantum chaotic attractors are generally of the same family as the classical attractors, we also find quantum attractors with no classical counterpart. Contrary to the standard wisdom, the correspondence limit can thus be the most difficult to achieve for certain classically chaotic systems. These phenomena occur in experimentally accessible regimes.

## Introduction

Lyapunov exponents quantify the sensitive dependence on initial conditions of dynamical systems. When the largest Lyapunov exponent *λ* is positive, the system is algorithmically complex^1^. That is, *λ* > 0 indicates that the growth in error from the predicted trajectory is exponentially sensitive to initial error. For such systems we must describe initial conditions with greater and greater precision to enable us to predict their behavior for longer times, unlike for systems with *λ* ≤ 0^1^. This is a remarkable qualitative and not merely quantitative distinction between chaotic and non-chaotic physical systems, characterized by the Lyapunov exponent or its information-theoretic equivalents. A system can go between chaotic and non-chaotic behavior with a small change of a physical parameter even in the absence of a qualitative change in the system.

That the dynamical behavior can be so distinctly different in algorithmic complexity matters greatly in understanding the behavior of nonlinear quantum systems near the classical limit. Schrodinger’s equation does not allow for exponential sensitivity to initial conditions. At the same time, *λ* > 0 for the classical limit of only certain nonlinear systems and not others. Since the world is fundamentally quantal, and the classical equations are an approximation valid only in the limit of a very large system, this leads to the important question: What is the difference between quantum systems that reflects the deep difference between classically chaotic and classically regular behavior? This question drove the detailed exploration of quantum spectra and eigenfunctions as signatures of quantum chaos. In the process, multiple other criteria have been defined attempting to find a mapping from algorithmic complexity (or chaos) in the classical limit of a quantum system to some characteristic of the quantum system itself. All of these are termed ‘quantum chaology’ in the absence of dynamical quantum chaos^2,3^. Absent a single criterion across quantum and classical systems it is particularly unclear how to characterize complexity for intermediately sized systems where it is not obvious that they are by either a quantum model or a classical model. How do we describe systems in the regime of semiclassical dynamics (usually studied under the name of ‘quantum-classical correspondence’)?

In fact, it was recognized early that general properties (expectation values of operators, behavior of wavefunctions, etc) for closed quantum systems show singularities in the classical limit if that limit is chaotic. A critical step in resolving the question of why we do not see such singular effects in nature was to understand that this was due to decoherence effects that smooth the quantum-classical transition (QCT)^4–11^. A further advance in understanding the behavior of systems in the semiclassical parameter regime (which was now established as arising from a combination of system size and environmental coupling) was the mapping of quantum stochastic trajectories formalisms to experimentally realizable behavior. Pioneering theoretical steps in this direction were the discovery of quantum chaotic Poincare sections^12,13^ and finally the explicit calculation of quantum *λ* > 0^14–17^. Calculations of quantum *λ* allow us to unify the understanding of chaos as algorithmic complexity across physical systems at all scales, and initial calculations also demonstrated that the chaos transitions smoothly to the classical limit for certain systems.

Such studies are also significantly practical since nonlinear quantum systems are important in quantum computing and simulating many-body systems; that is, nonlinearity is a resource for non-classicality^18^. Further, the existence of chaos also affects coherent control^19^, decoherence^4–11^, and quantum engineering generally. Current theory continues to examine how quantum trajectory chaos can be best identified^20,21^ and current experiments (some searching for dynamical quantum chaos) including those in nonlinear optomechanical^22^ and nano-electro-mechanical systems^23^ are in this regime.

In this paper, we investigate chaos for a paradigmatic system, the driven dissipative double-well quantum Duffing oscillator. We use parameters such that this system models experimentally accessible systems and explicitly accounts for the role of environmental effects arising either from thermal effects or measurements, yielding a standard nonlinear stochastic Schrodinger equation. This is a system well-studied previously^13,24–27^, including by two of the current authors. Most previous work including ours^25,27^ had focused on Poincare sections and time-series analysis. The studies of Lyapunov exponents in this system^24,26^ did not address or answer the question of how (or if) *λ* transitioned to the infinite size (classical) limit. This question is of particular interest for this system because the classical limit has dramatically different behaviors depending on small changes in the system-environment coupling strength Γ.

We study this system using both Γ and a dimensionless parameter *β*. The latter is effectively the inverse system size or scaled Planck’s constant (${\beta }^{2}\sim \hslash$), such that *β* → 0 describes the infinite size limit which is expected to behave classically, while *β* = 1 describes the extreme quantum regime where the uncertainty principle allows for roughly one quantum state to participate in the dynamics. Since the classical limiting behavior is strongly dependent on Γ, studying a range of Γ effectively allows us to study various different quantum-classical transitions. It is also helpful below to consider the quantity *K* = *λ* + Γ which we call the dynamical complexity of this system. While *λ* remains the diagnostic for chaos, *K* enables a simple accounting for the global effects of dissipation. In particular, *λ* = −Γ for all simple periodic orbits (POs) and since phase-space dissipatively contracts as Γ, we have that all POs have *K* = *λ* + Γ = 0. For this system *K* thus quantifies the dynamical complexity of an orbit relative to simple POs.

Our results are that

1. Broadly, *λ* for a quantum system is *decreasingly parametrically sensitive* to environmental coupling as *β* increases. That is, *λ* changes less for a quantum system as a function of Γ compared to the classical limiting behavior, and in particular does not follow the abrupt classical transitions between regularity and chaos as present in the classical limiting system as it transitions in and out of period-doubling cascades as function of Γ.
2. Consistent with this, the range of Γ displaying dynamical complexity *increases* with *β*.
3. When the classical limit is chaotic, *λ decreases* as *β* increases.
4. There are multiple regimes where *λ* is *non-monotonic* with *β*, including several remarkable examples where the system transitions from classical *λ* < 0 (no chaos) to quantum *λ* > 0 (chaos) as *β* increases.
5. Finally, we also find quantum chaotic attractors of a completely different character than any seen in the classical system.

These results on how *λ* behaves in the semiclassical regime as a function of *β*, Γ not only (a) contradict the current consensus^22,26^ that quantum effects supress chaos, and (b) contradict the intution that a quantum chaotic system must have a classically chaotic limit, but also (c) provide evidence that chaos may exist only in the semiclassical regime for certain systems although absent in either the classical limit or for the deep quantum regime, and in general (d) quantitatively demonstrate that the transition in behavior, and specifically the dynamical complexity between classical and quantum limits is a richly complex one, and non-monotonic in general. Our results are closely related to other recent work on the non-monotonic behavior of quantum chaos in the Duffing system where the parameter being varied is related to the measurement method^28^. All this further illustrates the varieties of behavior available in the ‘no-man’s land’ between systems described completely classically and systems described completely quantum-mechanically, with pragmatic consequences for devices working in this regime (nonlinear vibrational energy harvesters, for example).

Our paper starts with a quick overview of how the quantum Duffing system behaves as system size changes and an analysis of the classical limit behavior, before presenting the quantum results and our best understanding of the conditions under which this non-monotonicity may occur. We conclude with some observations about future directions.

## Model and Methods

### Quantum model and transition to classical model

We start by considering the transition of the quantum system to the classical limit. A general quantum system interacting with the environment is described by the Ito equation^29^

1 $$|d\psi \rangle =-\frac{i}{\hslash }\hat{H}|\psi \rangle dt+(\hat{L}-\langle \hat{L}\rangle )|\psi \rangle d\xi +(\langle {\hat{L}}^{\dagger }\rangle \hat{L}-\frac{1}{2}{\hat{L}}^{\dagger }\hat{L}-\frac{1}{2}\langle {\hat{L}}^{\dagger }\rangle \langle \hat{L}\rangle )|\psi \rangle dt$$

with the Hamiltonian $\hat{H}$ and the Lindblad operator $\hat{L}$ representing a zero-temperature Markovian environment^29^. *dξ* = *dξ*_*R*_ + *idξ*_*I*_ is a normalized complex differential random variable satisfying *M*(*dξ*) = 0; *M*(dξdξ^*^) = *dt* where *M*(⋅) denotes the mean over realizations. We can specialize this equation to the dimensionless^13,24,25^. Duffing oscillator using ${\hat{H}}_{\beta }={\hat{H}}_{D}+{\hat{H}}_{R}+{\hat{H}}_{ex}$ where ${\hat{H}}_{D}=\frac{1}{2}{\hat{P}}^{2}+\frac{{\beta }^{2}}{4}{\hat{Q}}^{4}-\frac{1}{2}{\hat{Q}}^{2},\,{\hat{H}}_{R}=\frac{{\rm{\Gamma }}}{2}(\hat{Q}\hat{P}+\hat{P}\hat{Q}),\,{\hat{H}}_{ex}=-\frac{g}{\beta }\hat{Q}\,\cos ({\rm{\Omega }}t)$ and the Lindblad $\hat{L}=\sqrt{{\rm{\Gamma }}}(\hat{Q}+i\hat{P})\mathrm{.}$ The quantum nonlinearity and dissipation both scale with Γ, via $\hat{L}$. *β* is dimensionless and is constructed from the system’s parameters of mass *m*, length *l*, and natural frequency *ω*_0_ as *β*^2^ = $\frac{\hslash }{m{l}^{2}{\omega }_{0}}$. It is an effective Planck’s constant determining the system’s degree of ‘quantumness’^13,24^. That is, larger *β* describe a smaller system needing fewer quantum states to describe the dynamics, and *β* → 0 is the classical limit. Recent analysis^23^ shows that nano-electro-mechanical systems are well described by this model with parameters of recent experiments within range of the phenomena we report.

The semiclassical equations for $\langle \hat{Q}\rangle ,\langle \hat{P}\rangle$ are also useful^24,30^. These centroid variables depend on second moment terms *σ*_*QQ*_, *σ*_*PP*_, *σ*_*PQ*_ where ${\sigma }_{AB}=\langle ({\hat{A}}^{\dagger }-{\langle \hat{A}\rangle }^{\ast })(\hat{B}-\langle \hat{B}\rangle )\rangle$. The various *σ* are themselves time dependent and depend on higher order terms like *σ*_*QQQ*_ etc. An infinite hierarchy^31^ of equations for higher moments obtains in general. Various approximations are possible^32^ and a truncation at second moments yields

2 $$dx=pdt+2\sqrt{{\rm{\Gamma }}}((\mu -\frac{1}{2})d{\xi }_{R}-Rd{\xi }_{I})$$

3 $$dp=(-{\beta }^{2}({x}^{3}+3\mu x)+x-2{\rm{\Gamma }}p+\frac{g}{\beta }cos(\omega t))dt+2\sqrt{{\rm{\Gamma }}}(Rd{\xi }_{R}-(\kappa -\frac{1}{2})d{\xi }_{I})$$

4 $$\frac{d\mu }{dt}=2R+2{\rm{\Gamma }}(\mu -{\mu }^{2}-{R}^{2}+\frac{1}{4})$$

5 $$\frac{d\kappa }{dt}=2R(-3{\beta }^{2}{x}^{2}+\mathrm{1)}+2{\rm{\Gamma }}(-\kappa -{\kappa }^{2}-{R}^{2}+\frac{1}{4})$$

6 $$\frac{dR}{dt}=\mu (-3{\beta }^{2}{x}^{2}+\mathrm{1)}+\kappa -2{\rm{\Gamma }}R(\mu +\kappa ),$$

where $\langle \hat{Q}\rangle \equiv x,\langle \hat{P}\rangle \equiv p\equiv \dot{x}$, *R* ≡ (1)/(2)(*σ*_*QP*_ + *σ*_*PQ*_), *σ*_*QQ*_ ≡ *μ* and *σ*_*PP*_ ≡ *κ*. This system can be understoon as that of the centroid *x*, *p* oscillator coupled to the *R*, *κ*, *μ* oscillator representing the spread variables.

As has been previously shown, as *β* → 0 the range of *x*, *p* increases as $\frac{1}{{\beta }}$ while the *σ* and *dξ* terms remain unchanged in scale. Thus, as *β* decreases, product terms like $\simeq \sigma d\xi$ quickly become relatively negligible, followed by the separate *σ*, *dξ* terms, representing respectively the influence of the spread variables and the fluctuating environment. Finally, for *β* sufficiently small we get behavior that is identical to (and hence can be represented by) the *β*-invariant equation

7 $$\ddot{x}+2{\rm{\Gamma }}\dot{x}+{\beta }^{2}{x}^{3}-x=\frac{g}{\beta }\,\cos ({\rm{\Omega }}t\mathrm{).}$$

That is, Eq. (7) is invariant under *β* → Λ*β*; *x* → $\frac{x}{{\rm{\Lambda }}}$ for any positive real Λ. It describes the large size (classical) limit of the quantum system, i.e. the dynamics of a classical particle identified with the quantum centroid. We note the recovery of the well-known fact^33^ that for a quantum system interacting with a zero-temperature environment via a non-Hermitian Lindblad there are no noise terms in the large size limit. That is, once a system is large enough, it is insensitive to quantum fluctuations. The classical limit equation can be understood describing a unit mass in a double-well potential, with dissipation Γ and a sinusoidal driving of amplitude *g* and frequency Ω (driving period *T* = 2*π*/Ω); it also represents the behavior of the center of mass of driven doubly-clamped beam.

This approach of recovering the classical dynamics as a limiting case of the quantum system by varying a single scaling parameter ensures that *all* the systems studied are experience the same (quantum) physics. It is only the length scale (or *β*) that determines the behavior seen. Further, the scaling of various terms with *β* is consistent with, and justifies the semiclassical truncation. The semiclassical accuracy is validated simply by comparing with the full quantum evolution. It is important to note the useful fact that for a given parameter set Γ, *g*, Ω, as we decrease *β*, once the semiclassics agree with the full quantum dynamics at some *β*_sc_ the accuracy only *improves* for *β* < *β*_sc_. Thus, there is always a minimum *β* below which the semiclassical dynamics are all we need to understand how the quantum system behaves differently from the classical one.

While the *β*_sc_ dependence on Γ, *g*, Ω is complicated, empirically ${\beta }_{{\rm{s}}{\rm{c}}}\ge {\beta }^{\ast }\simeq 0.01$ for the range of systems we report on in this paper. This is helpful, since we find empirically that the full quantum calculations scale exponentially in computational difficulty as *β* decreases (our numerics seemed roughly to go as exp(*β*^−3^)). We use two quantum implementations (the QSD library^29^ using a moving basis technique efficient at higher Γ and low *β*, and a fixed basis XMDS2^34^ method which performs better at lower Γ and higher *β*), along with semiclassics at even lower *β*, to cover the full range reported. For those systems for which we were able to validate the semiclassical calculations, we are able to do a more dense scan of parameters. In this paper all calculations reported for *β* ≤ 0.005 were performed semiclassically.

### Classical Duffing oscillator

**Figure 1:**
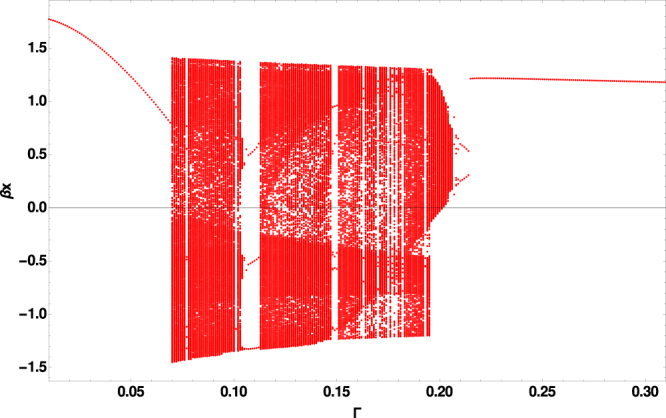
Classical bifurcation diagram as a function of coupling to environment (damping) Γ showing visually the transition from regular behavior to chaos as Γ increases from 0, and the transition back to regular behavior at high damping Γ > 0.205. Note the existence of high period regular behavior at particular Γ values within the chaotic regime.

We first review the behavior of the classical Duffing problem before reporting on the quantum results. In Fig. (1) we show the various dynamics that obtain as we vary Γ. To construct this bifurcation diagram we do the following: Pick a particular Γ and then initialize a trajectory inside one of the wells such that the energy < 0; we have verified that the behavior described below is independent of the specific location inside the wells. We then evolve the initial condition via Eq. (1) until *t* = 200*T* while discarding the transients (we choose that to be the first 10 driving cycles i.e. only keep results with *t* > 10*T*). During the post-transient evolution, we record *x* at *t* = *nT* for all *n*, and plot *all* the *x*(*t* = *nT*) results against the given value of Γ. Because we are recording *x* after every cycle of the driving, any PO will show up as a finite number of dots, and a chaotic trajectory which wanders densely in phase space will show as a solid vertical line. Finally we repeat this process by varying Γ. We have chosen non-zero Γ since Γ = 0 is the (singular) Hamiltonian limit, and is particularly problematic for the quantum system. We have restricted ourselves to results below Γ < 0.3 since the high damping makes the behavior trivial beyond that value. Unless otherwise stated, *g* = 0.3, Ω = 1 for the rest of the discussion.

If chaotic attractors persist as a function of Γ, the solid lines corresponding to chaos yield a solid band. Similiarly POs that persist yield simple curves. Also visible are periodic-doubling cascades and transitions to (and from) chaos with Γ. The embedded windows of regularity (e.g. at Γ = 0.110) within generally chaotic dynamics result from higher-order symmetries emerging in the dynamics. This behavior is generic in chaotic systems, though not always present^35^. We have seen (though do not report here in the paper) similar qualitative behavior if *g*, Ω are scanned in keeping with the literature^36^.

### Quantifying chaos and dynamical complexity

**Figure 2:**
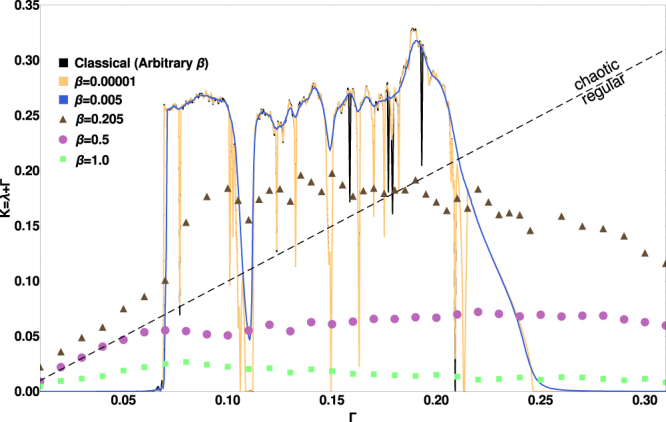
Complexity *K* = *λ* + Γ vs Γ for various *β*. Orbits are chaotic if and only if *K* is above the diagonal *K* = Γ line. Results are computed with full quantum mechanics for *β* ≥ 0.205 and with semiclassical equations for *β* ≤ 0.005. The results become independent of *β* for *β* < 0.000001 which is the expected classical limit. The semiclassical convergence to the classical is smooth. This is evidenced by the near-complete overlap of the *β* = 0.00001 curve with the classical curve, as well as the closeness of the *β* = 0.005 curve to the *β* = 0.00001 curve. The differences between the *β* = 0.005 and *β* = 0.00001 curves are visible only when the former does not follow the latter for all the dips in *K*, for example at Γ = 0.11 or Γ = 0.15. The difference between *β* = 0.00001 and the classical results are visible only in even narrower windows as a dark (classical) line dipping below the lighter *β* = 0.00001 curve for example at Γ = 0.19.

The dynamical complexity of this behavior is quantified by *K*, plotted against Γ in Fig. (2); To compute *λ*, a coherent state *ψ*_fid_ is initialized within one of the wells. A random small unitary kick is applied to *ψ*_fid_ to create *ψ*_per_. These wavefunctions are evolved via Eq. (2) with identical noise realizations and the behavior of the phase space distance between them yields $\lambda \equiv {\mathrm{lim}}_{t\to \infty }{\mathrm{lim}}_{{\rm{\Delta }}x\mathrm{(0),}{\rm{\Delta }}p\mathrm{(0)}\to 0}\frac{1}{t}{\mathrm{log}}_{2}(\sqrt{\frac{{({\rm{\Delta }}x(t))}^{2}+{({\rm{\Delta }}p(t))}^{2}}{{({\rm{\Delta }}x\mathrm{(0))}}^{2}+{({\rm{\Delta }}p\mathrm{(0))}}^{2}}})$ where ${\rm{\Delta }}x(t)={\langle \hat{Q}\rangle }_{{\psi }_{{\rm{fid}}}}-{\langle \hat{Q}\rangle }_{{\psi }_{{\rm{per}}}}$ and Δ*p*(*t*) is similarly defined.

**Figure 3:**
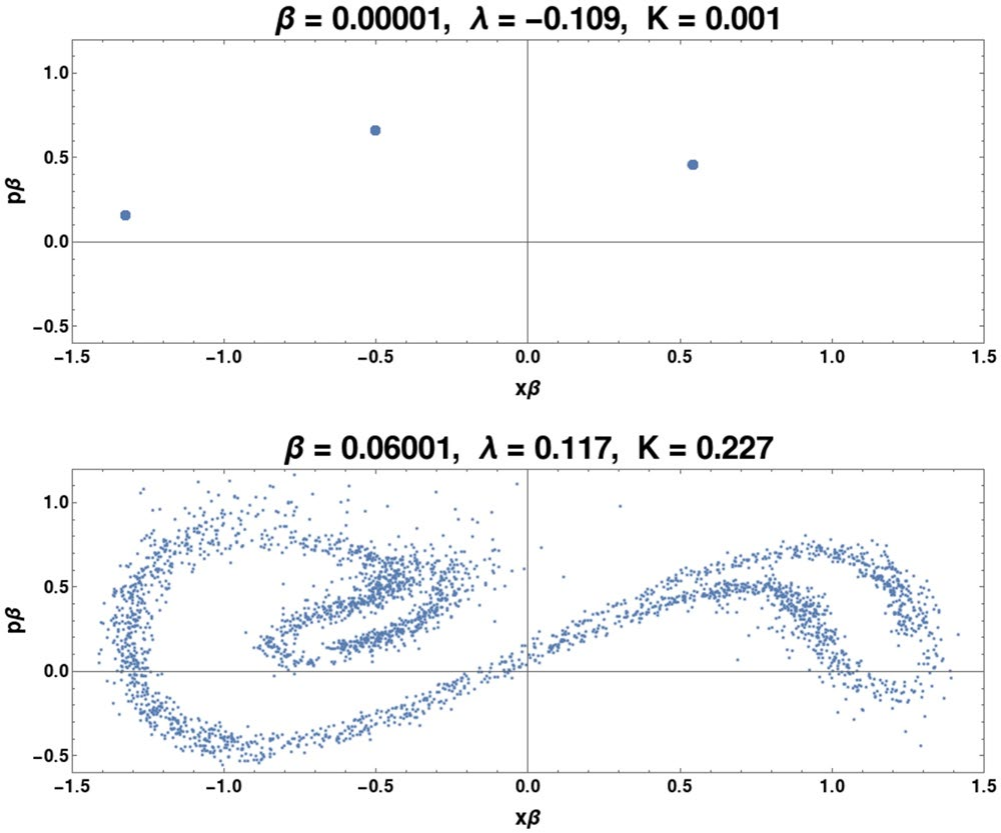
These are Poincare maps for Γ = 0.110 at two *β* values. For *β* = 0.00001, the system behavior is indistinguishable from classical behavior and we see a classical 3-period orbit. At *β* = 0.06001, notice the reemergence of the chaotic attractor along with *λ* > 0. At this relatively low *β*, the system transitions to quantum chaos with quantum effects destroying the phase-locking. The quantum chaotic attractor resembles the typical classical Duffing chaotic attractor.

**Figure 4:**
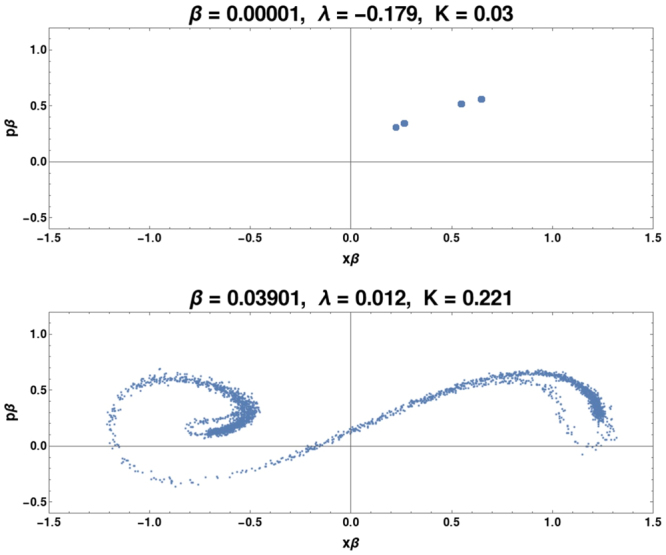
These are Poincare maps for Γ = 0.209 at two different *β* values. At *β* = 0.00001, the system is behaving essentially classically. At this *β* value, the system shows a single-well periodic behavior at high Γ. Notice that in the lower figure *λ* > 0, i.e., at relatively low *β*, quantum effects allow the system to cross the central potential barrier and we observe quantum chaotic behavior. This quantum chaotic attractor resembles the typical classical Duffing chaotic attractor.

**Figure 5:**
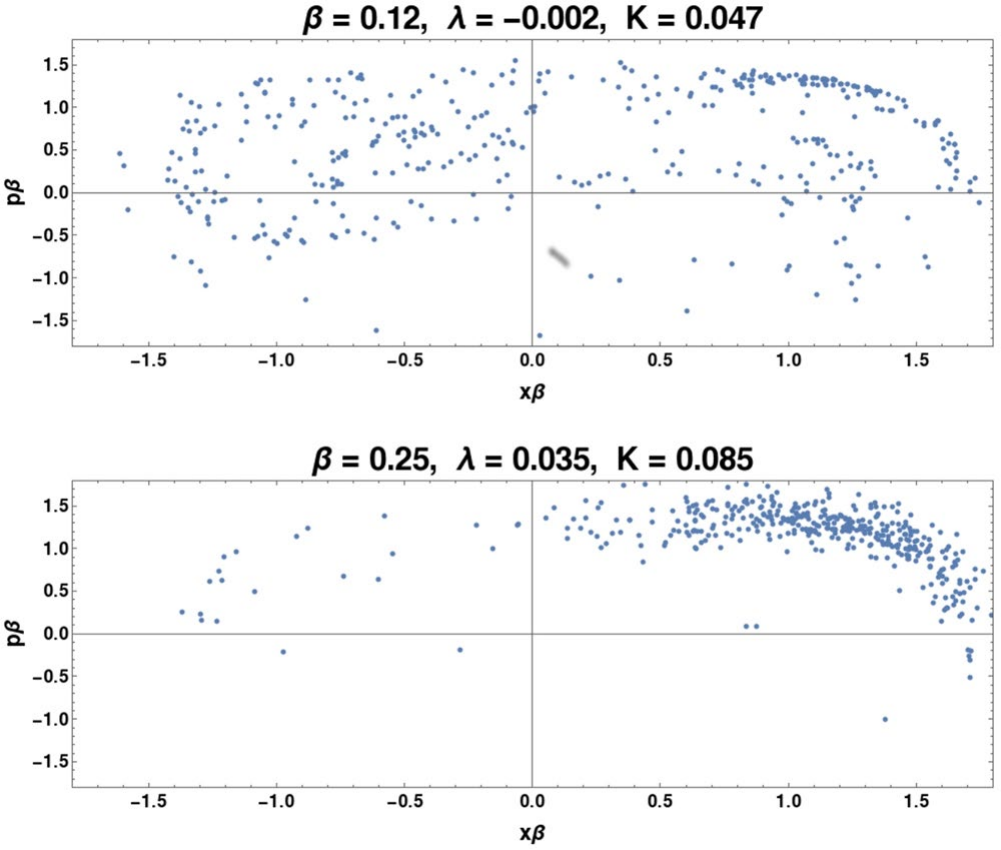
These are Poincare maps for Γ = 0.05 at two relatively large *β* values. As shown in Fig. (2), the classical behavior is regular at Γ = 0.05. Here the simple classical regular behavior persists for relatively large *β* values, as demonstrated in the Poincare map for *β* = 0.12, where *λ* < 0. As *β* is increased, a quantum chaotic attractor emerges, as demonstrated in the Poincare map for *β* = 0.25, where *λ* > 0. Not only is the system transitioning from classically regular to quantum chaotic behavior, as was the case in Fig. (3) and in Fig. (4), but also the quantum chaotic attractor is entirely unlike the typical Duffing classical attractor.

We use standard procedures to ensure convergence. As canonical^37^, instead of using zero initial distance we periodically reset *ψ*_per_ so that it remains within the linear deviation regime, and average over a large number of resets. The *λ* are averaged over 3000*T* and over *N*_*i*_ trajectories, each of which has a different noise realization. The standard error for *λ* decreases with *N*_*i*_ until it is within a few percent for a sufficiently large ${N}_{i}^{\ast }$. For the range of parameters reported at most ${N}_{i}^{\ast }=128$ trials were required to achieve the desired accuracy. For the special cases we discuss in detail and are presented in Figs (3, 4 and 5) we ran the simulations out to 10000*T* as an extra check, and have verified that all the results remain within a few percent of the results at 3000*T*. We have also verified that the results reported are independent of the precise choice of initial state within the wells. This is repeated for various *β*, and as *β* → 0 the initial conditions and their dynamics become identical to classical trajectories rendering this technique for calculating *λ* identical to the canonical technique^37^.

As is clear, this quantification of quantum dynamical chaos improves on all other approaches in being equivalent to the classical metric, as well as allowing the behavior to be mapped as a function of *β* so that we can study the intermediate regime and the approach to classical behavior as a limiting case. Calculations with *λ* defined in Hilbert space also yield similar behavior at intermediate and large *β* but do not yield the correct classical limits for small *β* and are not reported here. Orbits are chaotic if and only if *K* is above the Γ diagonal, i.e. if and only if *λ* > 0. A big advantage of considering *K* instead of *λ* is that it allows us, in this particular system, to use the behavior of *K* to see how the Duffing system switches between a family of chaotic attractors and regular behavior as a function of Γ, as we now discuss.

We focus first on *K* (and *λ*) as a function of Γ for the classical system as shown by the line labeled ‘classical’ in Fig. (2). The behavior of *K* for various quantum systems also shown there is discussed below. As Γ initially increases away from zero the classical orbits remain simple with *K* = 0. For Γ_1_ ≈ 0.06 there is an abrupt transition to complexity (*K* > 0 and *λ* > 0), which persists until Γ_2_ ≈ 0.210, except for many small windows of regularity (*λ* = −Γ, *K* = 0) most prominently around Γ ≈ 0.110 for example. Thus we see the system switching between POs and one family of chaotic attractors as we scan Γ. For Γ < Γ_1_ the global attractor is a PO that moves between the two wells, with the driving compensating for the dissipation. Γ_1_ marks when the dissipation is large enough such that the orbit does not always cross the central potential barrier. This can yield chaos: given two nearby orbits, one may cross the barrier while the other does not, leading to drastically different evolution. When Γ > Γ_2_ the high damping restricts the dynamics entirely to one of the wells, which does not yield complex behavior. Thus, the *K* > 0 ‘complexity hill’ arises from the dynamics interacting with the central potential barrier. This complexity hill marks a transition between simple behavior of differing symmetry.

Thus, classically simple (*K* = 0) dynamics corresponds to: (i) At low dissipation Γ < Γ_1_, trivial double-well POs, (ii) at high dissipation Γ > Γ_2_, trivial single-well POs, and (iii) within the complexity hill, at intermediate dissipation Γ_1_ < Γ < Γ_2_, high order POs in Γ windows of various sizes. A scan of Poincare sections at various Γ values shows that essentially the same chaotic attractor emerges for all chaotic dynamics. The POs that emerge at special Γ values do so due to a rare lining up of relevant time-scales (mode-locking of the dissipation with the driving frequency) such that the orbit closes onto itself. These POs are particularly sensitive to small perturbations, a fact which becomes of relevance as we consider quantum dynamics. This descriptive account of the dynamical complexity as a function of Γ for the classical system is quantified in more detail below when compared to the behavior of quantum systems.

## Results and Analysis

We start with a descriptive account of the behavior of quantum *λ* as a function of parameters before moving to a quantitative description. Again using *K* in Fig. (2) for clarity, we compare the classical results against the quantum behavior with *β*_1_ = 0.00001 (the near-classical limit), *β*_2_ = 0.005, *β*_3_ = 0.205, *β*_4_ = 0.5, and the deep quantum *β*_5_ = 1.0. These curves can be summarized according to the following observations:

- The quantum behavior broadly resembles the classical with a complexity hill at intermediate Γ. The difference from the classical behavior increases as the system becomes more quantum (as *β* increases).
- For any Γ, as *β* decreases, *K* starts low at *β* = 1.0 and increases at least through *β* ≈ 0.2.
- If the classical limit is chaotic, the classical values for *K* and *λ* are within a few percent of being the largest across all *β*, such that the classical system is the most chaotic.
- If the classical limit is *not chaotic K* and *λ* are non-monotonic with *β*. That is, there is always a quantum system more dynamically complex than the classical limit. This includes several examples where the quantum system is chaotic even when the classical system is regular. Such examples exist in all three Γ regimes with classical POs.

We turn to understanding the quantum results focusing on the general differences from the classical behavior. We start with a fixed *β* and consider the Γ dependence of *K*. Generically (e.g. for *β* = 0.205), *K* increases as Γ initially increases. This can be understood as arising from the fact that the quantum evolution depends nonlinearly on Γ via Eq. (2). As with the classical limit, *K* has a complexity hill, followed by a slower-than-classical decrease for larger Γ, such that quantum systems have a broader complexity hill. This can be attributed to quantum systems being able to cross the classical potential barrier. That is, even when the classical limiting behavior is confined to a single well, quantum effects which include an effective lowering of the potential as well as increased susceptibility to environmental fluctuations (to different degrees, depending on the value of *β*) allow the quantum system to move between the two wells, leading to greater dynamical complexity. Even at Γ ≈ 0.3 intermediate *β* systems have *K* greater than the classical system despite not being chaotic. This explains why previous work^25–27^ found evidence of complex time-series behavior at Γ = 0.3 for quantum systems for *β* = 0.3 even though the Hilbert space Lyapunov exponent was not appreciably greater than zero, and we find in fact a negative phase-space *λ*.

We now consider the *β* dependence of *K* for fixed Γ: At *β* ≈ 1.0 the dynamics are described by ≈2 quantum states, resulting in low dynamical complexity (low *K*, though not as low as classical POs which have *K* = 0). As *β* decreases from 1.0, the effective Hilbert space increases in size, and the system has greater dynamical complexity (increased *K*). This is borne out by our computations, where decreasing *β* increases the size of the basis set needed for convergent results. Also, as *β* decreases (system becomes more classical), the complexity hill becomes more prominent (*K* increases). This demonstrates the usual argument that classical systems are generally more complex than quantum systems. Finally, for small enough *β* the quantum results trace the classical curve, including the narrowest *K* = 0 windows. As *β* decreases it is accompanied by a decrease in the number of Γ windows where the classical and quantum results disagree. For *β* ≤ 10^−8^ the results are indistinguishable from the classically generated results.

We present here a quantitative abstraction of the behavior of *λ* which allows us to systematically distinguish and discuss the various ways in which quantum systems deviate from (and limit to) the well-known classical behavior. It is possible to represent *λ*(Γ, *β*) as

8 $$\lambda ({\rm{\Gamma }},\beta )={{\rm{\Pi }}}_{0}({{\rm{\Gamma }}}_{1},{{\rm{\Delta }}{\rm{\Gamma }}}_{0},\varepsilon )(K-{\rm{\Gamma }}\mathrm{)[1}-\sum _{{{\rm{\Gamma }}}_{i}}^{{N}_{w}}{R}_{i}{{\rm{\Pi }}}_{i}({{\rm{\Gamma }}}_{i},{{\rm{\Delta }}{\rm{\Gamma }}}_{i},{\varepsilon }_{i})],$$

where *β* dependence is implicit in the dependence of Γ_1_, ΔΓ_0_, *K* and all the *R*_*i*_, *ε*_*i*_ on *β*. The windowing function Π is the so-called “Planck-taper” window, a bump function widely used. It is smooth *C*^∞^ everywhere, but is exactly zero outside of a compact region, exactly 1 over an interval within that region, and varies smoothly and monotonically between those limits. The tapering (setting the range over which the function is exactly 1) is controlled by the parameter *ε* with smaller values giving sharper transitions. The limiting case is the rectangular function

9 $${\rm{\Pi }}=(\begin{array}{cc}0 & {\rm{if}}\,{{\rm{\Gamma }} < {\rm{\Gamma }}}_{{i}}\\ 1 & {\rm{if}}\,{\rm{\Gamma }}\ge {{\rm{\Gamma }}}_{{i}}\\ 0 & {\rm{if}}\,{\rm{\Gamma }}\ge {{\rm{\Gamma }}}_{{i}}+{{\rm{\Delta }}{\rm{\Gamma }}}_{{i}}\end{array}$$

that is, it is non-zero only for Γ_*i*_ < Γ < Γ_*i*_ + ΔΓ_*i*_. This sum over windowing functions allows us to represent the fact that as *β* → 0 the classical system is chaotic only for the ‘complexity hill’ of the window Π_0_ defined by Γ_1_, ΔΓ_0_ where Γ_2_ − Γ_1_ = ΔΓ_0_. Further, within this overall window are embedded the Π_*i*_ windows of regularity with abrupt transitions between chaos and regularity (where (∂*λ*)/(∂Γ) spikes twice at each boundary). If a particular window does not peak to unity, this is captured by the value *R*_*i*_ < 1. If we rank-order the sum over these windows (with a total number of windows *N*_*w*_) such that it runs from the smallest to the largest window, it proves useful in considering the quantum behavior.

This complicated *λ*(Γ) function allows us to delineate the effect of increasing quantum effects with *β*, listed in the order in which they become noticeable as *β* increases away from the classical limit. The first is that the various windows become smoother as *β* increases with the *λ*(Γ) curve less able to follow all the dips and rises of the classical curve for higher *β*. Second, both the left and the right edges of the global window Γ_1_, Γ_2_ are broadened. Third, within those windows that are classically chaotic, there is an overall decrease of *λ* with *β* for a fixed Γ within a chaotic region. The three *β*-dependent effects can be modeled as follows

- Since increasing *β* smooths increasingly more classical windows of regularity which disappear in order of increasing ΔΓ, we have that *ε* = *ε*_*c*_ + *f*_1_(*β*), where *ε*_*c*_ is the classical value (for either the left or right value within a certain window) and *f*_1_(*β*) is an *increasing* function of *β*. A secondary and less important effect is that *R*_*i*_ can decrease with increasing *β*.
- The quantum broadening of the global Γ_1_ < Γ_2_ window is captured in (Γ_1_(*β*), ΔΓ_0_(*β*)) of the global Π_0_ window being a function of *β*, where Γ_1_ is a *decreasing* function of *β*, while ΔΓ_0_ is an *increasing* function of *β*. There is also an important smoothing effect in *ε*_0_(*β*)(*β*) increasing with *β* (as for the smaller windows).
- Finally *K*_*β*_ is a *decreasing* function of *β* starting at *K* for *β* → 0 and represents an overall decrease in degree of dynamical complexity and indeed in chaos as quantum effects are increased, although initially swamped by the first two effects.

The first effect implies that the more quantum a system is, the less *parametrically* sensitive to Γ it is compared to the classical system. This quantum smoothing of the *λ*(Γ) curve arises from quantum effects differentially affecting the regular and chaotic classical dynamics–that is, the addition of quantum terms to the classical dynamics does not significantly affect classical chaos but does destroy the delicate structure of the higher-order mode-locked classical POs. It also means that there are regimes where *λ*(*β*) is non-monotonic with *β*. Specifically this means that the system transitions from classical *λ* < 0 (no chaos) to quantum *λ* > 0 (chaos) as we consider systems of decreasing size (larger effective &planck;), as we describe below. In fact we also find quantum chaotic attractors of completely different character from any attractor we see in the classical system in a regime outside the Γ_1_ < Γ < Γ_2_ window.

We now focus on three different ‘anomalous’ transitions where *λ* goes from negative to positive (i.e. the system goes from regular to chaotic) as it becomes more quantal. These three cases are chosen as both (a) representing other such transitions at nearby Γ values and (b) being distinct from each other. In all three cases, we use semi-classical dynamics to guide our understanding of how the classical regularity yields to chaos as *β* increases. The transitions happen in the regime where the semiclassical calculations are either quantitatively valid (in the first two cases below) or qualitatively yield the same behavior (even if disagreeing quantitatively, in the third case below).

Recall that in the semiclassical regime the full system is the classical *x*,*p* nonlinear Duffing oscillator coupled to the dynamics of (*μ*, *κ*, *R*). This second oscillator increases the effective dimensionality of the system, and its effect on the dynamics increases with increasing *β*. The sensitivity of the system to environmental fluctuations also increases as *β* increases.

The first example is in the intermediate dissipation regime (e.g. Γ = 0.110) where the quantum attractors resemble the classical chaotic attractors at neighboring Γ values and have approximately the same *K*. These chaotic quantum attractors arise from classical regular behavior with higher-order POs, and emerge even for small *β*. An example of this kind of quantum attractor is shown in the Poincaré sections of Fig. (3), constructed by recording (*x*(*t*), *p*(*t*)) at *t* = *nT* for all *n* after transients. At Γ = 0.110 the regular essentially classical (*β* = 0.00001) behavior in Fig. (3) is destroyed by the quantum mechanics which ‘recovers’ the classically chaotic attractor, as is clear from comparing the figure at *β* = 0.06001 in Fig. (3) with the classical attractor in refs^13,24,25^.

For this first example, orbits with the delicate integrability of the high-order mode-locked classical POs in the two-dimensional *x*, *p* phase-space are destroyed by the coupling to the (*R*, *κ*, *μ*) oscillator and environmental fluctuations as *β* increases. This understanding is consistent with the observation that the narrowest regular windows–where the smallest change to the classical equations sends the system into chaos–are the most difficult to reproduce semiclassically or quantum mechanically (i.e. are destroyed for the smallest *β*).

The second example resembles the first in emerging at low *β* and recovering the same attractor as the classical dynamics. However, it occurs beyond the classical chaotic hill, at the relatively high dissipation Γ = 0.209. The Poincare sections are shown in Fig. (4). The difference from the situation at Γ = 0.110 is that here the classical regular trajectory (on the left) does not arise from mode-locking but from being dissipatively constrained to one well. As *β* increases quantum effects, that is, the 5–dimensional dynamics allowing energy to flow from the spread variables as well as the greater susceptibility to environmental fluctuations, allow the centroid to cross the classical potential barrier. It is not clear that this would be classified as tunneling at the lower *β* values studied since the increased sensitivity to fluctuations play an important role. This semiclassical ability to cross the barrier yields a quantum chaotic attractor resembling the classical attractors from smaller Γ values, with quantum *K* again approximating those of the classical attractors. In fact classical bifurcation digrams constructed as a function of Γ starting from Γ = 0.209 and with Γ decreasing resemble those constructed as a function of *β* (also starting with Γ = 0.209) but with increasing *β*.

Not all anomalous chaos restores the classical attractor. A third regime, where the mechanism seems to be fundamentally different, is at low Γ < 0.06 where the classically system displays ‘simple’ double-well regular orbits. Here the transition to quantum chaos is at fairly large *β*(≈0.2) and the attractors that emerge do not exist classically. That this is not ‘recovered’ classical chaos but is uniquely quantal is manifest in the visual evidence of the Poincare sections (Fig. (5)) not resembling the other attractors, and also because both *K*, *λ* differ substantially the values for the chaotic attractors (classical and quantum) in the other regimes. Also, compared to the classical behavior where *λ* < 0, the attractor contracts in phase-space for the chaotic system (*λ* > 0) and despite low dissipation is largely in one well. In the other two cases (Γ = 0.110, 0.209), the attractor increases in range in phase-space at the onset of quantum chaos.

At Γ = 0.05 this happens for *β* greater than ≈0.12. At this *β* the semiclassical Eqs (5–7) for *μ*, *κ*, *R* are not accurate but are still instructive in deducing the mechanism leading to the chaotic dynamics. First considering *β* near 0 and Γ ≈ 0, we see that *μ*, *κ*, *R* evolve linearly, and have a constant of motion (*R*^2^ − *μκ*). For Γ = 0.05, the Γ terms are first order corrections in Eqs (3–7) and do not engender chaos. However, as *β* increases, the *β*^2^*x*^2^ term nonlinearly couples Eqs (3,4) with Eqs (5–7) and breaks the integrability. This effect–and the effect of the environmental fluctuation terms–also appear for the other anomalous chaos cases discussed above, but in this particular case *β* is much larger when chaos occurs, and the nonlinearity is structurally different, as visible in the attractor. The system is thus not restoring the classical attractor but creates a wholly new attractor. This analysis using semiclassics is empirically justified by the fact that a semiclassical attractor computed at these values qualitatively resembles the attractor computed from the full quantum equations (and neither resembles any attractor found in the classical dynamics). Irrespective, this is uniquely quantum trajectory chaos, without a corresponding classical attractor.

## Concluding discussion

In summary, we find that the quantum-to-classical transition of the dynamical complexity for open nonlinear quantum systems is substantially more complicated than the current understanding that quantum effects cause classically chaos to transition to quantum regularity. In particular, if the classical system (*β* → 0) has regular behavior corresponding to high-order dynamical symmetries, we need very large length scales (very small *β*) to recover correspondence. That is, ironically contrary to the standard wisdom^2^, the correspondence limit can be the most extreme for certain classically regular systems. Further, the break point (*β*_sc_) depends on the chaos in complicated ways worth exploring. We have seen examples (though we do not include these results here) where the semiclassics works better for a system with classical chaos compared to a system that is classically regular, contrary to standard intuition.

How general is this behavior? The ingredients for this wealth of behavior and anomalous or non-monotonic transistions are sufficient quantum nonlinearity (size of Lindblad terms), and intermediate *β* (so the Hilbert space is large enough to not be too simple but *β* is not so small as to track classical regularity). We have shown above that even with a single model system this yields a transition from three different kinds of classical regularity to quantum chaos in very different parameter regimes. We thus expect that such behaviors should be generic and we have indeed observed similar non-monotonic and anomalous behavior in very different parameter regimes (though we do not include those results here).

How this behavior depends on the specific choice of Lindblad operator (that is, the shape of the transition depending on the form of the environmental coupling) is another intriguing question currently being investigated^28^. Exploring both experimentally and theoretically the multi-parameter landscape of system properties and environmental coupling is expected to uncover interesting and useful quantum nonlinear behavior.
